# Control of CCR5 Cell-Surface Targeting by the PRAF2 Gatekeeper

**DOI:** 10.3390/ijms242417438

**Published:** 2023-12-13

**Authors:** Elisa Da Silva, Mark G. H. Scott, Hervé Enslen, Stefano Marullo

**Affiliations:** CNRS, INSERM, Institut Cochin, Université Paris Cité, F-75014 Paris, France; elisa.da-silva@inserm.fr (E.D.S.); mark.scott@inserm.fr (M.G.H.S.); herve.enslen@inserm.fr (H.E.)

**Keywords:** G protein-coupled receptor, cell trafficking, endoplasmic reticulum, ERES, escort protein, gatekeeper, biosensor, BRET

## Abstract

The cell-surface targeting of neo-synthesized G protein-coupled receptors (GPCRs) involves the recruitment of receptors into COPII vesicles budding at endoplasmic reticulum exit sites (ERESs). This process is regulated for some GPCRs by escort proteins, which facilitate their export, or by gatekeepers that retain the receptors in the ER. PRAF2, an ER-resident four trans- membrane domain protein with cytoplasmic extremities, operates as a gatekeeper for the GB1 protomer of the heterodimeric GABA_B_ receptor, interacting with a tandem di-leucine/RXR retention motif in the carboxyterminal tail of GB1. PRAF2 was also reported to interact in a two-hybrid screen with a peptide corresponding to the carboxyterminal tail of the chemokine receptor CCR5 despite the absence of RXR motifs in its sequence. Using a bioluminescence resonance energy transfer (BRET)-based subcellular localization system, we found that PRAF2 inhibits, in a concentration-dependent manner, the plasma membrane export of CCR5. BRET-based proximity assays and Co-IP experiments demonstrated that PRAF2/CCR5 interaction does not require the presence of a receptor carboxyterminal tail and involves instead the transmembrane domains of both proteins. The mutation of the potential di-leucine/RXR motif contained in the third intracellular loop of CCR5 does not affect PRAF2-mediated retention. It instead impairs the cell-surface export of CCR5 by inhibiting CCR5’s interaction with its private escort protein, CD4. PRAF2 and CD4 thus display opposite roles on the cell-surface export of CCR5, with PRAF2 inhibiting and CD4 promoting this process, likely operating at the level of CCR5 recruitment into COPII vesicles, which leave the ER.

## 1. Introduction

The cell-surface concentration of polytopic plasma membrane proteins, such as receptors, transporters, and ion channels, is controlled by various mechanisms, including constitutive and induced endocytosis [1] and the delivery of neo-synthesized proteins from the endoplasmic reticulum (ER) to the plasma membrane via the Golgi apparatus. Transport from the ER to the Golgi complex involves the selective capture of membrane proteins to be exported to the cell surface into COPII carrier vesicles [2] located in specific localized areas, known as ER exit sites (or ERESs) [3].

There is experimental evidence that G protein-coupled receptors (GPCRs) need to be loaded into COPII vesicles to exit the ER [4]. GPCRs can be actively captured by COPII via specific motifs and/or direct interactions with COPII components that, in turn, affect their export dynamics [5]. Whereas in many cases the vesicle recruitment and export of correctly folded GPCRs seems to occur by default as part of fluid and membrane bulk flow [2], other receptors require oligomerization and/or interaction with specific chaperones or escort proteins to leave the ER [6]. In addition, several GPCRs are retained within intracellular compartments, limiting or preventing their export to the cell surface. In most cases, GPCR retention is caused by mutations and is associated with accelerated proteasome degradation, leading to pathological effects [7]. However, some non-mutated GPCRs are also selectively retained within intracellular compartments and are released to ERESs upon cellular signaling [8] for a fine-tuning of their biological function.

A prototypical example of GPCR physiological intracellular retention is the GB1 subunit of the GABA_B_ receptor, the metabotropic receptor of the principal inhibitory neuromediator. Functional GABA_B_ receptors are hetero-dimers constituted of GB1 and GB2 protomers [9,10,11]. GB1 contains the GABA binding site [9] but fails to reach the cell surface when expressed in heterologous systems or overexpressed in neurons [12]. GB1 contains a tandem di-leucine motif and an arginine-based signal (RXR) within its carboxyterminal tail, which, together, cause its ER retention [13,14]. GB2 couples to G-proteins [15,16] and can reach the cell surface in the absence of GB1 as a functionally inactive homo-dimer [17]. The shielding of the GB1 retention signal via a coiled-coil interaction with the carboxyterminal of GB2 allows the hetero-dimer to reach the cell surface [13]. We reported that Prenylated Rab Acceptor Family member 2 (PRAF2), a ubiquitous membrane-associated protein [18] particularly abundant in the ER [19], was the gatekeeper that retains GB1 in the ER via the recognition of its RXR-di-leucine signal [20]. PRAF2 tightly controls cell-surface GABA_B_ density in vitro and in vivo [20]. PRAF2 was subsequently found to interact with both wild-type and mutant cystic fibrosis (CF) transmembrane conductance regulator (CFTR) on a stoichiometric basis, preventing the access of newly synthesized cargo to ERESs. The PRAF2-CFTR interaction involves a specific RXR-di-leucine motif located in the first nucleotide-binding domain of the transporter [21].

The GABA_B_ receptor is a class-C GPCR, with a particular specificity for its unique heterodimeric organization. Until now, it has not been established whether PRAF2 can regulate the export of other GPCRs. The chemokine receptor CCR5 is a good Class-A GPCR candidate to test for potential regulation by PRAF2 for several reasons. Indeed, PRAF2 was originally isolated in a two-hybrid screen using the C tail of the chemokine receptor CCR5, and its overexpression in reconstituted cell systems correlates with the reduced expression of the receptor [22]. In addition to its physiological role in chemotaxis and in the effector functions of T lymphocytes, macrophages, and dendritic cells, CCR5 is the main co-receptor for HIV-1 cell entry, in association with CD4 [23]. The cell-surface association between CCR5 and CD4 is a prerequisite for gp120 viral protein binding [24,25]. In resting primary T lymphocytes, a proportion of the CCR5 receptor is stored in intracellular compartments [26], and its interaction with CD4 in the ER facilitates its export to the cell surface, with CD4 acting as a “private” escort protein for CCR5 [27]. The physiological intracellular retention of CCR5 has been corroborated by experiments on THP-1 monocytes in vitro, showing that cell adhesion on fibronectin-coated slides for 10 min was sufficient to increase surface CCR5 five-fold, independently of CD4 [28]. Finally, some natural mutants of CCR5 lacking the C-terminal tail are completely retained in the ER, preventing HIV-1 infection in humans [29]. These data indicate that CCR5 retention in the ER is an important regulated phenomenon, suggesting a potential mechanistic role for PRAF2 in this context. However, the lack of PRAF2 binding motifs identified for GB1 and CFTR in the C-tail of CCR5 likely indicates different modes of interaction/retention.

## 2. Results

**Figure 1:**
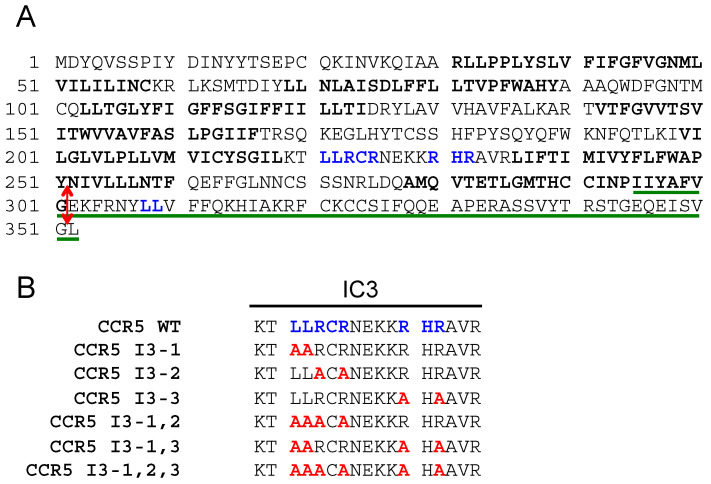
Potential PRAF2-interacting motifs in the sequence of human CCR5. (**A**) Protein sequence of human CCR5; the seven transmembrane domains are in bold. Putative PRAF2 di-leucine (LL) and arginine-based interaction motifs (RXR) present in the CCR5 third intra-cytoplasmic loop and C-terminus are in blue. The sequence of the CCR5 peptide used in the two-hybrid screen, which identified the interaction of CCR5 with PRAF2, is underlined. The red bi-directional arrow follows the last amino acid residue of the CCR5-ΔCter mutant. (**B**) Nomenclature of CCR5 third intracellular loop (IC3) LL/RXR motif mutants investigated in the study (mutations are in red).

The protein sequence of human CCR5 is shown in Figure 1A. Di-leucine (LL) motifs and arginine-based motifs (RXR) present in the third cytoplasmic loop and in the C-terminal tail of the receptor are in blue. FS299, a natural CCR5 mutant principally found in Asia, consists of a single base pair deletion, causing a frame shift near the end of the seventh transmembrane domain (amino acid residue position 299). A premature termination occurs, causing the absence of the intracellular C-terminal domain. This mutant is poorly expressed on the cell surface and retained within intracellular compartments [29,30]. We introduced a stop codon after glycine 301 to produce the CCR5-ΔCter mutant, which was also similarly retained within intracellular compartments as the FS299 natural mutant [27]. The sequence of the C-terminal CCR5 (295–352) peptide used as bait in the two-hybrid screen, which identified PRAF2 as a CCR5-interacting partner [22], is underlined in green.

**Figure 2:**
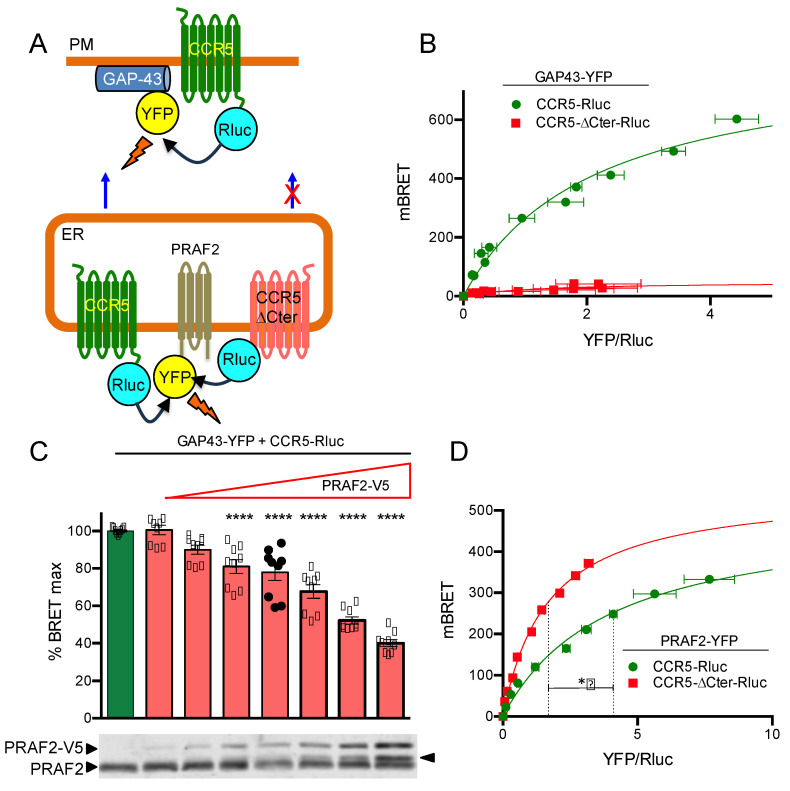
Overexpression of PRAF2 reduces CCR5 export to the cell surface, probably by retaining the receptor in the ER. (**A**) Diagrams of BRET-based assays. Top: biosensor to measure CCR5 export to the cell surface. Bottom: BRET assay to monitor PRAF2/CCR5 proximity. Wild-type CCR5 is exported to the cell surface, only part of it in proximity to the BRET acceptor fused to PRAF2. CCR5 missing the carboxy-terminal tail (CCR5-ΔCter) is totally retained in the ER and can be fully saturated by PRAF2-YFP. (**B**) BRET saturation curves (see Methods) obtained by co-expressing CCR5-Rluc or CCR5 ΔCter-Rluc in HEK-293 cells with increasing concentrations of GAP43-YFP BRET acceptor. Mean values ± SEM are shown (data compiled from 3 independent experiments in triplicate). For CCR5-Rluc, the BRET_max_ (BRET value at saturation) was 825.5 ± 28.5, and the BRET_50_ (the YFP/Rluc ratio at 50% saturation) was 2.1 ± 0.15. (**C**) Monitoring cell-surface CCR5 in the presence of increasing concentrations of PRAF2. BRET experiments were conducted with constant amounts of CCR5-Rluc and GAP43-YFP and increasing concentrations of PRAF2-V5 (0–1.4 µg of transfected DNA). Mean values ± SEM are shown (data from 3 independent experiments in triplicate. ****: *p* ≤ 0.0001). *p*-values were calculated using one-way ANOVA followed by Dunnett’s multiple comparison test with CCR5-Rluc in the absence of PRAF2-V5. The immunoblot at the bottom shows the actual expression of PRAF2-V5 relative to the endogenous PRAF2. Note that, at the highest concentrations, a fraction of PRAF2-V5 is partially degraded (arrowhead). Since the MW of the cleavage product migrates above the endogenous PRAF2, the cleavage probably occurs within the tag, generating a fragment, which includes an entire functional PRAF2. (**D**) BRET experiments comparing the proximity of CCR5-Rluc and CCR5 ΔCter-Rluc to PRAF2-YFP. BRET_max_ values: 498.2 ± 20.7 (R^2^ = 0.9; 88 dof) for CCR5 and 552.4 ± 14.5 (R^2^ = 0.99; 88 dof) for CCR5 ΔCter-Rluc, *p* = 0.16 (ns). BRET_50_ values: 4.0 ± 0.3 and 1.7 ± 0.1 for CCR5-Rluc and CCR5 ΔCter-Rluc, respectively, *p* ≤ 0.05 *; dof: degrees of freedom.

A proximity assay with a bioluminescence resonance energy transfer (BRET)-based biosensor was used to monitor the cell-surface targeting of CCR5 (Figure 2A). GAP43 (growth-associated protein 43) is a neuronal cytoplasmic protein that is attached to the inner face of the plasma membrane via a dual palmitoylation sequence on cysteine residues 3 and 4. The first 20 amino acid residues of GAP43, fused upstream of the yellow fluorescent protein (YFP), were used as an energy transfer acceptor, whereas wild-type or mutant CCR5 fused upstream of *Renilla* luciferase was the BRET donor. Upon co-transfection of the relevant plasmids in HEK-293 cells, CCR5 exported to the cell surface in proximity to GAP43-YFP can produce a BRET signal. BRET saturation curves (Figure 2B) were generated by increasing the concentrations of GAP43-YFP in the presence of constant, equivalent amounts of CCR5-Rluc or CCR5-ΔCter-Rluc, which were controlled by their luciferase activity. A hyperbolic curve that reached saturation was obtained with CCR5-Rluc, indicative of its targeting of the cell surface. In contrast, low levels of bystander (linear) BRET were obtained with CCR5-ΔCter-Rluc, consistent with its reported almost exclusive intracellular retention [29].

PRAF2 has been reported to play the role of gatekeeper [31], capable of physiologically retaining, on a stoichiometric basis, wild-type GBR1 and CFTR in the ER [20,21]. The possible control of CCR5 export by PRAF2 was investigated by measuring the amount of CCR5 reaching the cell surface in the presence of increasing concentrations of PRAF2 with the biosensor used above (Figure 2C). Supporting this hypothesis, the BRET signal obtained decreased as a function of increasing concentrations of exogenous epitope-tagged PRAF2 (PRAF2-V5). The highest amount of PRAF2-V5, which appeared about two-fold higher than endogenous PRAF2 in an immunoblot (lower panes, Figure 2C), was associated with about 60% reduction in surface CCR5. To document the potential direct interaction of PRAF2 with CCR5 and CCR5-ΔCter, additional BRET-based proximity experiments were performed using PRAF2-YFP as a BRET acceptor (Figure 2A,D). In this configuration, hyperbolic curves were obtained from both donors (Figure 2D). Interestingly, significantly lower BRET_50_ values were measured for CCR5-ΔCter-Rluc compared with CCR5-Rluc, indicating the higher propensity of the former to be in proximity to PRAF2-YFP. Thus, the possible higher affinity of PRAF2 for CCR5-ΔCter might contribute to the greater retention of this mutant. The reduced maximal BRET (BRET_max_) obtained with CCR5-Rluc compared with CCR5-ΔCter-Rluc is due to the fact that the pool of CCR5-Rluc, having reached the cell surface, cannot transfer any energy to the ER-associated PRAF2-YFP.

**Figure 3:**
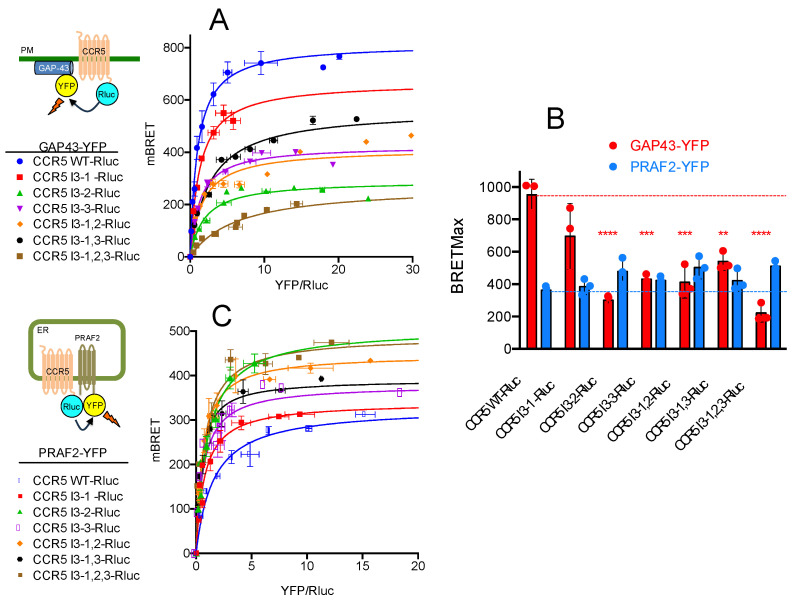
Effect of disrupting LL and RXR motifs in the third intracellular loop of CCR5 on surface export and PRAF2 proximity. (**A**) Comparison of plasma membrane targeting of CCR5 and CCR5 mutants (all HA-tagged and fused to Rluc; see Figure 1B for nomenclature) using the BRET biosensor described in Figure 2. Mean values ± SEM are shown (data from 3 independent experiments in triplicate). Calculated BRET_max_ values: 966.0 ± 20.5 (R^2^ = 0.98; 70 dof) for CCR5-RLuc; 668.5 ± 26.9 (R^2^ = 0.92; 70 dof) for CCR5 I3-1-Rluc; 294.9 ± 5.2 (R^2^ = 0.97; 70 dof) for CCR5 I3-2-Rluc; 425.3 ± 7.6 (R^2^ = 0.97; 70 dof) for CCR5 I3-3-Rluc; 397.4 ± 13.8 (R^2^ = 0.89; 70 dof) for CCR5 I3-1,2-Rluc; 538.0 ± 15.2 (R^2^ = 0.95; 70 dof) for CCR5 I3-1,3-Rluc and 223.1 ± 12.7 (R^2^ = 0.93; 70 dof) for CCR5 I3-1,2,3-Rluc (statistical analysis in (**B**)). BRET_50_ values (2.4 ± 0.15; 1.3 ± 0.15; 2.1 ± 0.13; 1.4 ± 0.1; 1.7 ± 0.2; 2.3 ± 0.2 and 4.7 ± 0.6, following the same order) were not different. (**B**) Comparison of BRET_max_ values calculated with GAP43-YFP (red histograms, curves in (**A**)) or PRAF2-YFP (blue histograms, curves in **C**) as BRET acceptors. *p*-values calculated using one-way ANOVA followed by Tukey’s multiple comparison test between CCR5-Rluc and indicated mutants are summarized as follows: ** *p* ≤ 0.01; *** *p* ≤ 0.001; **** *p* ≤ 0.0001; the absence of asterisks indicates a nonsignificant difference. (**C**) BRET proximity assays of CCR5 and CCR5 mutants with PRAF2 (as in Figure 2D). Mean values ± SEM are shown (data from 3 independent experiments in triplicate). BRET_max_ values (same order as in (**A**)) were not significantly different: 369.1 ± 9.5 (R^2^ = 0.97; 67 dof); 386.3 ± 9.3 (R^2^ = 0.97; 67 dof); 489.7 ± 14.2 (R^2^ = 0.94; 64 dof); 450.4 ± 17.5 (R^2^ = 0.93; 61 dof); 509.0 ± 13.8 (R^2^ = 0.94; 70 dof); 434.9 ± 11.9 (R^2^ = 0.94; 67 dof); 518.3 ± 13.1 (R^2^ = 0.94; 64 dof). Corresponding BRET_50_ values were also not different: 2.4 ± 0.2; 1.4 ± 0.1; 1.0 ± 0.1; 1.5 ± 1.2; 1.3 ± 0.1; 0.8 ± 0.1; 1.0 ± 0.1.

Since PRAF2 was originally identified as an interacting partner of the C-terminal tail of CCR5 [22], which is absent in the CCR5-ΔCter mutant, additional domain(s) of interaction with PRAF2 likely exist in the CCR5 sequence. In particular, PRAF2 was reported to interact with tandem LL RXR motifs present in the GB1 C-terminal tail [20] and in the CFTR nucleotide-binding domain, NBD1 [21]. Similar motifs appear in the third intracellular loop (I3) of CCR5. We, therefore, investigated whether the LL and RXR motifs of CCR5-I3 might participate in interactions with PRAF2. A series of CCR5 mutants were made, in which these motifs were substituted individually or in association (Figure 1B) and tested for their cell-surface export (Figure 3A). The substitution of each of these motifs variably affected the amount of CCR5-Rluc that reached the cell surface, reflected by the maximal BRET signal obtained with GAP43-YFP as an acceptor. Whereas the substitution of the di-leucine motif alone (CCR5 I3-1-Rluc) did not significantly modify the BRET_max_ value (Figure 3A,B), all other mutations inhibited the surface export of CCR5, with the substitution of the first RXR motif of the I3 loop accounting alone for about 70% of the inhibition (see mutant CCR5 I3-2-Rluc). In parallel experiments measuring BRET between CCR5-Rluc or CCR5-Rluc mutants and PRAF2-YFP, neither the BRET_max_ nor BRET_50_ values were statistically different between the CCR5 and CCR5 mutants (Figure 3B,C), indicating that the LL and RXR motifs in the I3 of CCR5 are not critical for the receptor interaction with PRAF2.

**Figure 4:**
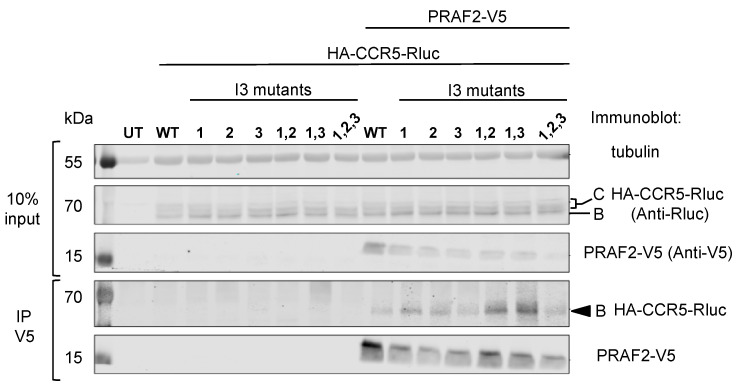
Co-immunoprecipitation experiments of wild-type and mutant CCR5 fused to Rluc by PRAF2-V5. HEK-293 cells were co-transfected with plasmids coding for HA-tagged and Rluc-tagged CCR5 or I3 mutants (described in Figure 1 and Figure 3) and a plasmid coding for PRAF2-V5 or an empty vector (*n* = 3). After immunoprecipitation with the anti-V5 antibody, co-immunoprecipitated material was separated with PAGE, blotted, and probed with the indicated antibodies. Input is shown on the top part of the panel. Band B corresponds to a core-glycosylated receptor in the ER, and Band C corresponds to an additionally glycosylated receptor in the Golgi or on the cell surface.

Co-immunoprecipitation experiments (Figure 4) confirmed that PRAF2-V5 could interact with CCR5-Rluc and with all tested mutants, including CCR5 I3-1,2,3-Rluc, in which all di-leucine and arginine-based motifs were substituted.

**Figure 5:**
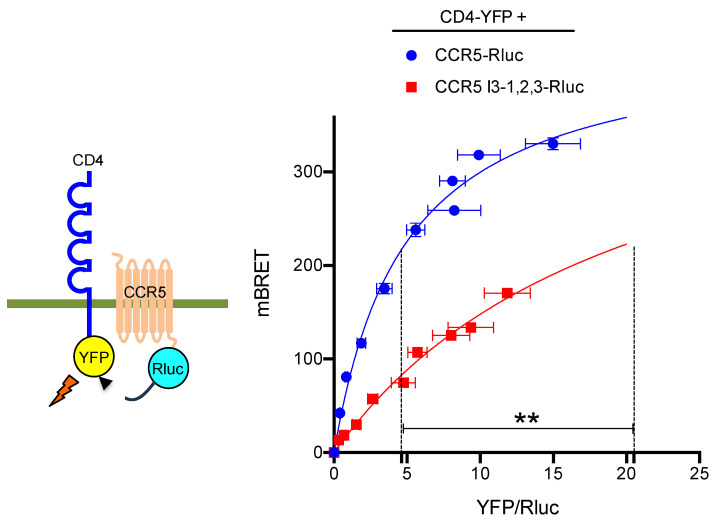
BRET saturation experiments between CCR5 and CD4. Constant, equivalent amounts of CCR5-Rluc and CCR5 I3-1,2,3-Rluc were co-expressed with increasing concentrations of the CD4-YFP BRET acceptor. Mean values ± SEM are shown; data were compiled from 4 independent experiments, each performed in triplicate. BRET_max_ values: 445.2 ± 11.1 (R^2^ = 0.97; 118 dof) for CCR5-Rluc and 451.7 ± 49.7 (R^2^ = 0.96; 116 dof) for CCR5 I3-1,2,3-Rluc, *p* = 0.8, ns. BRET_50_ values: 4.9 ± 0.3 for CCR5-Rluc and 20.5 ± 3.2 for CCR5 I3-1,2,3-Rluc, ** *p* = 0.0175. *p*-values were calculated using an unpaired t-test.

The massive retention observed in the CCR5 I3-1,2,3-Rluc mutant (Figure 3A, brown curve) might be due to its export-incompetent conformation. Indeed, previous studies have shown that CCR5 forms constitutive dimers [31] and adopts distinct conformations, some of them allowing CCR5 dimers to be targeted at the cell surface [32]. The HIV co-receptor CD4 has been reported to enhance CCR5 surface delivery in primary T lymphocytes or when co-expressed in reconstituted cell systems [27]. CD4-CCR5 interaction occurs within intracellular compartments, suggesting that CD4 might stabilize a conformation that allows CCR5 to enter the export pathway. Based on this paradigm, we used CD4 as a conformational probe for CCR5 and CCR5 I3-1,2,3 in a BRET assay (Figure 5). Confirming previous BRET-based studies of CCR5-CD4 interaction performed with inverted donor and acceptor pairs [27], the hyperbolic saturation of CCR5-Rluc was achieved with increasing concentrations of CD4-YFP. Using CCR5 I3-1,2,3-Rluc as a BRET donor, saturation was also achieved, but with a much higher BRET_50_, indicative of the markedly reduced capacity of CD4 to associate with the CCR5 mutant. This result is consistent with either the reduced affinity of CD4 for CCR5 I3-1,2,3 or some segregation of CCR5 I3-1,2,3 out of the ER-exiting pathway, where CD4 is physiologically located.

**Figure 6:**
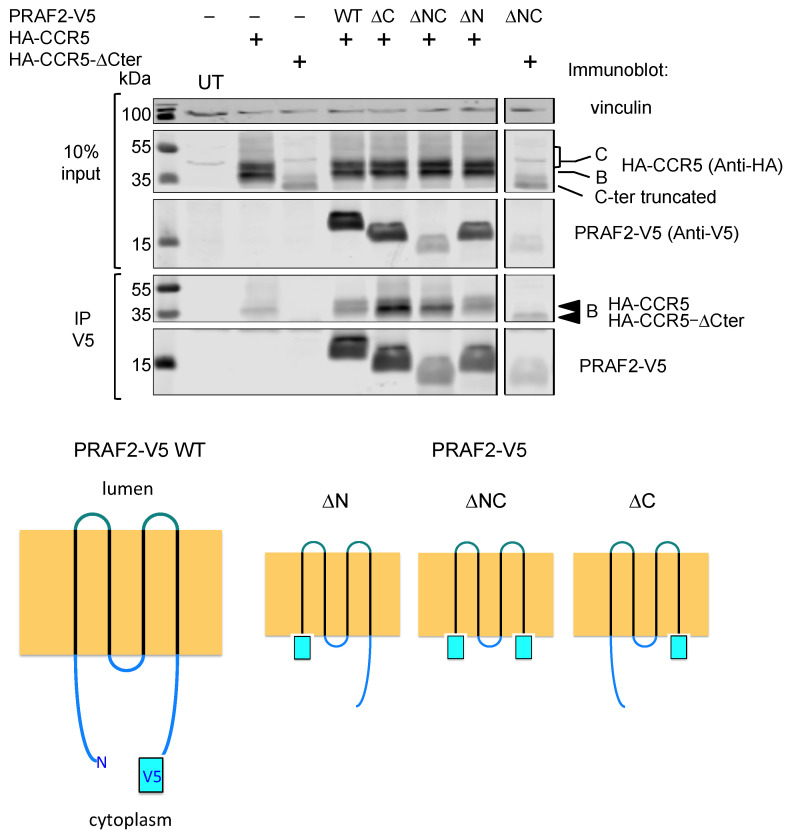
PRAF2 domains interacting with CCR5. HEK-293 cells were transfected with plasmids coding for HA-CCR5 or HA-CCR5-ΔCter in the presence or absence of plasmids coding for wild-type or mutant PRAF2-V5, displaying the indicated deletions (*n* = 3). After immunoprecipitation with the anti-V5 antibody, co-immunoprecipitated material was separated with PAGE, blotted, and probed with the indicated antibodies. The input is shown on the top part of the panel. Band B corresponds to the core-glycosylated receptor in the ER, and Band C corresponds to an additionally glycosylated receptor in the Golgi or on the cell surface. Bottom cartoon: V5-epitope-tagged wild-type PRAF2 (PRAF2-V5) and V5-tagged PRAF2 mutants deleted from the C-terminus (ΔC), N-terminus (ΔN), or both extremities (ΔNC) used in co-immunoprecipitation experiments.

The observation that the largest intracellular loop and the C-terminus of CCR5 were not essential for the interaction with PRAF2 raised the hypothesis that the transmembrane domains of the receptor and PRAF2 might instead constitute their principal interface. Consistently, it has been reported that the transmembrane domains of CCR5, TM5, and TM6 in particular are predominantly involved in the dimeric organization of the receptor, which is required for targeting CCR5 to the plasma membrane [32]. V5-epitope-tagged PRAF2 mutants were constructed, lacking either the N- or C-terminal intra-cytoplasmic region or both. These mutants were compared with wild-type PRAF2-V5 for their interactions with CCR5 and CCR5-ΔCter in co-immunoprecipitation experiments (Figure 6). PRAF2 and all tested truncated mutants, including the one lacking both cytoplasmic regions, could co-immunoprecipitate CCR5. Moreover, the latter mutant could also co-immunoprecipitate CCR5-ΔCter. Together with the observation above that the LL and RXR motifs of the I3 are not involved in receptor interactions with PRAF2 (Figure 3 and Figure 4), these data indicate that CCR5 and PRAF2 most likely interact via their TM domains.

## 3. Discussion

This study adds CCR5 to the range of plasma membrane polytopic proteins, whose cell-surface targeting is regulated by PRAF2. As for GB1 and CFTR, CCR5 retention is dependent on PRAF2 concentration. Previous mapping of endogenous PRAF2 distribution revealed a marked variability in PRAF2 expression in the brain, with PRAF2 being virtually absent in some areas, whereas other areas displayed very strong PRAF2 expression [19]. Moreover, PRAF2 overexpression has been reported in multiple cancers [18,33]. A 60% reduction in CCR5 expression for a two-fold enhancement of PRAF2 levels over basal values suggests the “physiological” regulation of CCR5 function through the modulation of PRAF2 concentration. Future studies will evaluate this hypothesis by measuring the regulation of endogenous PRAF2 expression in CCR5-expressing cells under different conditions of maturation or stimulation.

Inconsistent with the two-hybrid screen, which led to the identification of PRAF2 as a CCR5 interaction partner and regulator [22], the deletion of the CCR5 C-tail totally inhibited the cell-surface targeting of the receptor and enhanced its propensity to associate with PRAF2. PRAF2 interaction might thus mechanistically participate in the protection against HIV infection of individuals carrying mutant CCR5 receptors with truncated carboxy-terminal tails [29,30]. The substitutions of the LL and RXR motifs of the I3 failed to enhance the cell-surface export of CCR5 but impaired it instead. A maximal decrease in plasma membrane targeting was observed in CCR5 I3-1,2,3-Rluc, despite its propensity to associate with PRAF2-YFP, which was not significantly changed in BRET experiments, and its conserved co-immunoprecipitation with PRAF2-V5. Consequently, in contrast to what has been established for GB1 and CFTR, the interaction between CCR5 and PRAF2 likely involves their transmembrane domains instead of specific tandem LL-RXR motifs.

The HIV receptor CD4 was found to be constitutively associated with CCR5 in the plasma membrane of various cell types [24,34,35]. It has been proposed that interactions mediating this association involve the extracellular globular domain of CD4 (the first two Ig domains) and the second extracellular regions of CCR5, even in the absence of gp120 or any other receptor-specific ligands [25,34,36,37]. Some studies have shown the allosteric CD4-dependent regulation of the binding and signaling properties of CCR5 [36]. Moreover, other investigations have indicated that CD4 already interacts with CCR5 in the ER and plays the role of a private escort protein for this receptor, promoting its cell-surface targeting [27]. Here, we found that the substitution of the di-leucine and RXR motifs of the third intracellular loop of CCR5 markedly impaired its propensity for interactions with CD4 in BRET experiments, providing a possible explanation for the markedly perturbed plasma membrane localization of the CCR5 I3-1,2,3-Rluc mutant, which is perhaps due to conformational rearrangement.

In a recent study, an enzyme-catalyzed proximity labeling experiment using the Turbo ID approach, aimed at characterizing the PRAF2 interactome in the ER, showed the enrichment of proteins involved in “vesicular trafficking”, with the highest enrichment being for COPII-coated vesicle components [21]. PRAF2 was found to have a preferential proximity to the Sec machinery, including the Sec23/24 heterodimer involved in SNARE and cargo molecule selection [38], which is indicative of possible control over proteins transported to the cell surface near ERESs, from which COPII-vesicles bud. High-resolution fluorescence microscopy studies confirmed the proximity of PRAF2 to a significant fraction of COPII vesicles, marked by an anti-Sec31 (a COPII component) antibody without superposition, supporting a model where PRAF2 retains cargo proteins immediately upstream of their loading into vesicles leaving the ERES [21]. Interestingly, a mass-spectrometry-based analysis of macrophage proteins interacting with CD4 identified several proteins composing COPII-vesicles or regulating their fission from ER membranes [39]. Sec23/24 was included in this group, suggesting that CD4 might mediate the recruitment of CCR5 into COPII vesicles by bridging the receptor and the Sec23/24 cargo receptor.

In conclusion, PRAF2, likely by interacting via its TM domains with those of CCR5, inhibits its cell-surface export. In cells that express (endogenous or exogenous) CD4, CCR5 establishes an interaction with it involving its extracellular domain and its I3. The I3-mediated interaction with CD4 might be either direct or via a specific conformation stabilized by the intact I3. Physiologically, PRAF2 and CD4 have opposite roles in the same process, inhibiting or promoting the recruitment of CCR5 into COPII vesicles. It remains to be established if these regulating molecules act on the same or on distinct pools of vesicles leaving the ERES.

## 4. Materials and Methods

### 4.1. Cell Culture

Human Embryonic Kidney 293 (HEK-293) cells (ATCC^®^, Database name: Cellosaurus, Resource Identification Initiative: HEK293 (RRID:CVCL_0045)) were maintained in Dulbecco’s modified Eagle’s medium (DMEM; Gibco) supplemented with 10% FBS (Sigma) and 1% Penicillin–Streptomycin (Gibco) at 37 °C in a 5% CO_2_ humidified chamber.

### 4.2. Site-Directed Mutagenesis and DNA Constructs

The CCR5-Rluc construct was described previously in [32]. The construction of HA-CCR5-ΔCter-Rluc, PRAF2-YFP, and PRAF2-V5 plasmids was previously described in [20]. PRAF2-V5-, ΔN-, ΔN-, and ΔNC-truncated mutants were prepared via PCR amplification as follows: for ΔN, the forward primer included a new ATG immediately before the first amino-acid residue of TM1. For ΔC, the reverse primer included a stop codon after the last amino-acid residue of TM4. These primers were used together to generate the coding region of ΔNC.

The Quick-Change Site-Directed Mutagenesis Kit (Agilent) was used to perform mutagenesis following the manufacturer’s protocol, and all mutated plasmids were verified via DNA sequencing. The substitution of LL and RXR motifs by alanine residues in the third intracellular loop of CCR5 was achieved using this procedure. HA-CCR5-ΔCter was obtained from HA-CCR5-ΔCter-Rluc via the insertion in the CCR5 coding region of a STOP codon, immediately after the sequence coding for the glycine at position 301.

### 4.3. Antibodies and Reagents

The following monoclonal antibodies (mAb) or polyclonal antibodies were used for immunoprecipitation or immunoblotting experiments: anti-JM4/PRAF2 rabbit polyclonal antibody (ab53113; Abcam, Amsterdam, Netherlands), anti-V5 mouse monoclonal antibody (R960-25; Thermo Fischer, Villebon-sur-Yvette, France), anti-Rluc mouse monoclonal antibody (MAB 4410-I, Roche, Saint-Quentin Fallavier, France), anti-β-Tubulin rabbit monoclonal antibody (mAb #2128; Cell Signaling, Saint-Cyr l’Ecole, France), anti-Vinculin mouse monoclonal antibody (V9131; Sigma, Saint-Quentin Fallavier, France), and anti-HA rabbit monoclonal antibody (mAb #3724; Cell Signaling, Saint-Cyr l’Ecole, France). The following secondary antibodies were used in Western blot experiments: IR Dye^®^ 680 RD Donkey anti-Rabbit antibody (926–68073; Li-cor, Bad Homburg, Germany), IR Dye^®^ 800 CW Donkey anti-Rabbit (926–32213; Li-cor, Bad Homburg, Germany), IR Dye^®^ 800 CW Donkey anti-Mouse (925–32212; Li-cor, Bad Homburg, Germany), and IR Dye^®^ 680 RD Donkey anti-Mouse (925–68072; Li-cor, Bad Homburg, Germany).

The following reagents were used: Dulbecco’s phosphate-buffered saline (14190–094; Gibco, Villebon-sur-Yvette, France), Hanks’s balanced salt solution (14025–050; Gibco, Villebon-sur-Yvette, France), EcoTransfect (ET11000; OZBiosciences, Marseille, France), coelenterazine h (R3078b; Interchim, Montluçon, France), cOmplete EDTA-free protease tablets (05056489001; Roche, Saint-Quentin Fallavier, France), Protein G Sepharose 4 Fast Flow (17061801; Fischer Scientific, Illkirch, France), and Pierce™ BCA protein assay (23225; Thermo Fisher, Villebon-sur-Yvette, France).

### 4.4. Transfections

The indicated plasmids were transiently transfected in HEK-293 cells using the EcoTransfect (OZBiosciences, Marseille, France) transfection reagent following the manufacturer’s protocol.

### 4.5. Immunoprecipitation and Immunoblotting

For immunoprecipitation experiments, cells were washed twice and detached in PBS 1X. Cells were centrifuged at 1700× *g* at 4 °C for 4 min and then suspended in a lysis buffer containing 50 mM of HEPES (pH 7.4, adjusted with NaOH), 250 mM of NaCl, 2 mM of EDTA, 0.5% (*v*/*v*) Nonidet P-40, 10% (*v*/*v*) glycerol, and cOmplete protease inhibitor mix. Lysates were cleared via centrifugation at 18,000× *g* at 4 °C for 15 min. Cell lysates were pre-cleared with protein G beads for 3 h at 4 °C and then incubated with anti-V5 antibody (ThermoFisher, R960-25) overnight at 4 °C. Protein G beads were pre-incubated for 3–5 h at 4 °C in bovine serum albumin (BSA). Immune complexes were collected the following day by incubating them with protein G beads for 3 h at 4 °C. The beads were subsequently washed three times and eluted in 2X sample loading buffer prepared from a 5X stock (250 mM of Tris 0.75 M, pH 6.8; 10% SDS; 50% glycerol; 0.1% bromophenol blue; 10% 2 mercaptoethanol) at 37 °C for 15 min. The eluted samples were further analyzed via immunoblot.

For immunoblotting, the cells were lysed in the same lysis buffer (50 mM of HEPES (pH 7.4, adjusted with NaOH), 250 mM of NaCl, 2 mM of EDTA, 0.5% (*v*/*v*) Nonidet P-40, 10% (*v*/*v*) glycerol, and cOmplete protease inhibitor mix). Lysates were cleared via centrifugation at 18,000× *g* at 4 °C for 15 min and eluted in 1X sample loading buffer.

The total amount of protein was measured using the Pierce™ BCA protein assay (Thermo Fischer, 23225), and 25–50 µg or 500–1000 µg of protein for immunoblotting or immunoprecipitation, respectively, was separated via SDS–polyacrylamide gel electrophoresis. The samples were loaded in a 12% gel to separate CCR5 (≈35–55 kDa or ≈70–100 kDa when fused to Rluc) and PRAF2 (≈10–20 kDa). Separated proteins were transferred to the nitrocellulose membrane. The membranes were incubated in 5% skimmed milk in Tris-buffered saline containing 0.1% Tween-20 (TBST) for 1 h at room temperature, followed by incubation with the respective primary antibodies (dilution, 1:1000) at 4 °C overnight. The next day, the membranes were incubated with appropriate secondary antibodies from Li-cor for 1 h at room temperature, and labeled material was visualized/analyzed with the Odyssey CLx Imager.

### 4.6. BRET Saturation Assays

The BRET procedure was fully described previously in [40]. Briefly, HEK-293 cells were transfected with a constant amount of donor plasmid and increasing amounts of acceptor plasmid; the total amount of DNA was kept constant with empty vector DNA. Cells were washed in PBS and detached in HBSS. Cells were then plated in 96-well plates in triplicates, and coelenterazine h (5 µM) was added. The luminescence (filter 485 ± 10 nm) and fluorescence (filter 530 ± 12.5 nm) signals were measured simultaneously in a Mithras fluorescence–luminescence detector (Mithras2 LB 943 Multimode Reader). The calculated BRET ratio is the fluorescence signal over the luminescence signal, and the specific BRET was obtained by subtracting the signal from cells expressing the donor alone. The specific BRET was then multiplied by 1000 to be expressed as a milli-Bret. Specific BRET values were then plotted as a function of [(YFP−YFP0)/Rluc], where YFP is the fluorescence measured after excitation at 480 nm; YFP0 is the background fluorescence measured in cells not expressing the BRET acceptor; and Rluc is the luminescence measured after the coelenterazine h addition. The data were analyzed with GraphPad Prism with a nonlinear regression equation assuming a single binding site, and the BRET_max_ and BRET_50_ were calculated.

### 4.7. Quantifications and Statistical Analysis

For the statistical analysis of BRET curves, the one-site-specific binding model was compared using the extra sum of squares F Test for BRET_max_ and BRET_50_. The null hypothesis was that BRET_max_ and BRET_50_ were statistically the same in each data set. The null hypothesis was rejected if the p-value was less than 0.05. The exact p-value was reported for each experiment. All data are expressed as mean ± SEM. Data sets were compared with one-way ANOVA, and multiple comparison tests (Dunnett’s or Tukey’s) were performed with GraphPad Prism (Prism v9.5.0-525).

## Data Availability

All original data are available upon request.
